# Impairment of value-based decision-making in morphine-dependent rats is partly related to neural connectivity between the anterior cingulate cortex and basolateral amygdala

**DOI:** 10.17179/excli2023-6442

**Published:** 2024-01-03

**Authors:** Zahra Fatahi, Mohammad Fatahi, Abbas Haghparast

**Affiliations:** 1 Neuroscience Research Center, Shahid Beheshti University of Medical Sciences, School of Medicine, Tehran, Iran; 2 School of Electrical and Computer Engineering, College of Engineering, University of Tehran, Tehran 1439957131 Iran; 3 School of Cognitive Sciences, Institute for Research in Fundamental Sciences, Tehran, Iran; 4 Department of Basic Sciences, Iranian Academy of Medical Sciences, Tehran, Iran

**Keywords:** effort-based decision-making, basolateral amygdala, anterior cingulate cortex, neural synchronization, field potential recording, morphine dependence

## Abstract

Previous studies have established that the amygdala specifically the basolateral amygdala (BLA), has a fundamental role in decision-making. The present study aimed to investigate functional and neural synchronization between the BLA and anterior cingulate cortex (ACC) while making effort-choice decisions regarding pre-morphine dependence and morphine dependence times. A T-maze decision-making task with a differential outlay (great vs. small effort) and benefit (great vs. small reward) was done, and local field potentials from the BLA and ACC were assessed simultaneously. Results illustrated that in pre-morphine dependence time, when the animals made great reward/great effort decisions, there was a neural synchronization between both regions in beta and gamma frequency bands; and also, in delta, theta, beta, and gamma frequencies while expending effort and climbing the barrier. However, in morphine-dependent rats, during low reward/low effort choice and also during expending low effort, there was just a weak neural coherence in gamma frequency. Besides, there was neural synchronization in theta, beta, and gamma frequencies during reaching great reward in pre-morphine dependence time. Nevertheless, during reaching low reward in morphine dependence time, there was a weaker coherence in beta and gamma compared to pre-morphine dependence. These findings showed that functional and neural coherence between the BLA and ACC has a fundamental role in making the effort-based decision and expending effort. Preference for low reward/low effort, and decrease in expending effort in morphine-dependent rats is partly associated with the changes in the neural coherence between the BLA and ACC.

## Introduction

Making decisions is a common function that requires the evaluation of risks and remunerations related to different selections available. While deciding between two goods introduced differently, individuals choose based on exertion to obtain remuneration, amount of result, and a chance of a win (Morgado et al., 2014[32]). Cost-benefit decision-making leads to maximize rewarding results by choosing functions resulting in the best subjective cost (Adams et al., 2012[1]). Subjective cost is determined by evaluating the rewarding properties in light of the values that must be endured to obtain that result (Green et al., 2015[13]). The amygdala is a critical brain region in recognition of learning to anticipate future punishments based on predictive cues and communicates between expected value and the stimuli (Karen et al., 2006[21]). This area is one of the regions implicated in decision-making and has been determined to play an essential role in integrating efforts and remuneration information to support the best efficiency (Floresco and Ghods-Sharifi, 2007[10]; Hauber and Sommer, 2009[17]). Reportedly, the basolateral amygdala (BLA), anterior cingulate cortex (ACC), and nucleus accumbens core interact in regulating effort-based decision-making (Hart et al., 2018[15]), and dopaminergic circuits of these areas are essential in mediating this form of decision (Kristina et al., 2012[24]). These regions form a neural pathway that facilitates presumptive steps in the decision-making procedure, containing assessing existing alternatives, choice and implementation of a behavior, and processing of the result (Floresco et al., 2008[11]; Lane et al., 2010[25]). 

Opioid receptors are distributed widely in the human brain, and this system regulates affective processing, including pain, pleasure, and reward (Onge and Floresco, 2009[33]; Steenbergen et al., 2019[38]). It is shown that the µ-opioid receptor encodes motivation and preference for high-value rewards and controls distinction and assessment in the food, sexual and social domains (Onge and Floresco, 2009[33]). Chronic morphine administration has long-lasting effects on choice behavior (Eppolito et al., 2013[4]), and chronic opiate use is connected with enhancing impulsivity. It was revealed that acute morphine administration could enhance impulsivity in nondependent rats (Harvey-Lewis and Franklin, 2015[16]), and effort- or delay-based decision-making following presentation to morphine was impaired (Fatahi et al., 2020[7][8][9]). However, that is it not yet known how coherence between the BLA and ACC either guide or respond to decisions and how morphine would disrupt this.

Previous studies in our laboratory demonstrated neural connectivity between the ACC and orbitofrontal cortex during different forms of decision-making, including effort- and delay-based decision-making (Fatahi et al., 2018[6]; Fatahi et al., 2020[5]). Studies also revealed that morphine dependence disrupted effort- and delay-choice decision-making such that animals selected low benefit without effort instead of high benefit with the physical endeavor (Fatahi et al., 2020[7][8][9]). Hence, the present study assumed that disrupting effort-choice decision-making in morphine-dependent rats might be associated with altering the neural connection between the BLA and ACC. The current study investigates how the BLA and ACC neurons are synchronized and connected, while decision-making in morphine-dependent rats is determined by effort. For this purpose, this study recorded the local field potential (LFP) activities from both the BLA and ACC in pre-morphine dependence and morphine-dependence times while the effort-based decision-making was running.

## Materials and Methods

### Animals

In this study, male adult Wistar rats (Pasture Institute, Tehran, Iran) weighing 230-270 g were housed separately in a 12/12 h light-dark cycle and temperature-controlled environment (22 ± 2 °C). Initial body weights (85-90 % of the free-feeding weight) were adjusted at the beginning of the behavior, and then about 6-12 g per week was gained in a controlled manner. The water was existing ad libitum. Guide for the care and use of Laboratory Animals (National Institutes of Health Publication No. 80- 23, revised 1996) was used employed for all the experiments. Furthermore, the approval was given by the Institutional Ethics Committee of the Shahid Beheshti University of Medical Sciences (IR.SBMU.PHNS.REC.1398.136), Tehran, Iran, to the experiments.

### Apparatus

T-maze includes a beginning arm and two goal arms (right and left arms, length of 60 cm, width of 10 cm, and height of 40 cm for each arm). The food wells containing rewards, are provided at the end of goal arms, and their diameter is 3 cm. The T-maze is made of polyvinyl chloride, PVC (Fatahi et al., 2018[6]; Kermani et al., 2018[22]). Barriers (mesh wire, three-dimensional triangular) were placed in the middle of the high-reward goal arm to make effort-based decision-making. Differential heights (10, 20, and 30 cm) were assigned to the barriers to represent different physical cost and effort types during the process. The animal had to climb over a barrier to get great reward (Supplementary Figure 1A).

Five infrared sensors (IR) were embedded (two of them were at the inception of great reward and/or low reward arms, two of them were at the inception of reward wells, one of them was at the end of the beginning arm). Once the rat reached the end of the beginning arm and broke the infrared sensor, a sonic signal played from a broadcaster at the termination of the great reward arm to specify the great reward arm (in half of the trials, the great reward arm was on the left and the right for the other half). As soon as, the animals had decided in favor of the arm with the large reward and/or the arm with the small, they broke the infrared sensor at the beginning of the target arm. The time between broking two infrared sensors (at the end of beginning arm, and at the beginning of goal arm) was about 500 ms, and local field potential recording at this time was analyzed to investigate neural synchronization during effort-based decision-making (Supplementary Figure 1A). In addition, 500 ms after interruption, the infrared sensor at the beginning of the target arms was analyzed to assess neural coherence during effort and ascent from the barrier. 

Besides, while rats fractured infrared sensor at the beginning of the reward well (Supplementary Figure 1A), 500 msec before and 500 msec after that were assessed to determine neural coherence during reaching a reward. The movement sequences of the rats were recorded on video and provided with electrophysiological recordings. 

### Behavioral training

The training of the rats was done by putting rats in the two arms of the maze with unalike expenses (small or great) and costs (great or low effort) to perform T-maze decision-making tasks. The training process was almost the same as the procedure explained before (Fatahi et al., 2020[7]; Denk et al., 2005[3]). The handling of rats was done to make rats familiar with human contact every day before the training for a week. After putting rats on a limited feeding program, they were transferred to T-maze after receiving 85-90 % of their free feeding.

**Habituation step:** First, the animals were placed at the outset of T-maze (three rats simultaneously), and for 20 minutes, rats were free to seek throughout the maze. In the next three days, the animals checked the T-maze while there was abundant reward in both food wells of the goal arms (45 mg food reward, Formula A/ I; P. J. Noyes, Lancaster, NH).

**Discrimination step:** Once the familiarization phase is complete, the animals learn to distinguish between high-rewarded (six-part food rewards) and low-rewarded (two-part food rewards) target arms. Discrimination training takes place in three phases:

1. The great rewarded arm had six pieces of rewards and the low rewarded arm contained two pieces. The animals were on the start arm, and the large reward arm was emphasized by a sound signal. Doors were open, the animals were free to access both goal arms and get the reward from both arms. This step performed ten times a day by each rat for three days. 

2. In order for the rats to learn difference between two arms, one of the target arms was closed on each trial and the rats were forced to enter only one of the target arms. That is to say, there were ten “force” trials every day. In five trials animals forced to access just to low rewarded arm, and forced to access the great rewarded arm for the rest half.

3. In the third phase, two goal arms were open, and the rats were permitted to choose each of goal arms. When they chose one of the arms, that arm would be closed and the animals were allowed to gather food only from the same arm. There were ten “choice” trials every day with two minutes gap between trials, and all were done sequentially. The training extended until rats chose the great rewarded arm in 80 % for three sequential days.

**Barrier step:** To boost the physical effort expenses of the great reward, as soon as the animals got the average amount of 80 % for great reward, a 10 cm high obstacle (constructed of wire mesh) was placed in the intermediate of the great reward arm. As the same way, in this step, after a few days of training and getting over 80 % (average of great reward choice) for three following days, the barrier's height reached 30 cm at the end of the training process (with 10 cm steps) (Denk et al., 2005[3]; Supplementary Figure 1a).

### Morphine dependence protocol

In morphine dependence procedure, the animals received morphine (Temad Company, Tehran, Iran; 10 mg/kg; sc) twice per day (7:00 A.M. and 7:00 P.M.) for ten days (Miladi-Gorji et al., 2011[30]; Pu et al., 2002[36]). The animals were administrated with naloxone (2 mg/kg) to confirm the morphine dependence, and then behavioral manifestations of withdrawal (moving paws, head moves, stretches, grinding teeth, jumping, itching) were assessed for 30 min according to a modified version of the Gellet-Holtzman scale (Gellert and Holtzman, 1978[12]). Each day, three hours after morphine injection, each animal performed ten free-choice trials with an intertrial interval of 2 minutes to remember decision-making task until test day (10^th^ day of morphine dependence procedure). 

### General and surgical procedures

To implant the electrodes, injection of ketamine (100 mg/kg; intraperitoneally), and xylazine (8 mg/ kg) was done for anesthetizing. The animals were located and immobilized in a stereotaxic device (Stoelting Co., USA). Bipolar recording electrode wires (diameter: 0.005 in altogether ∼200 μm distance between the tips) was made by two rotated PFA-coated stainless-steel wires. The electrodes were located separately into the left ACC and BLA, and designation of coordinates was performed according to rat brain atlas (Paxinos and Watson, 1998[34]). For the BLA: 2.6 mm posterior to bregma, 4.8 mm lateral to the midline, and 8.5 mm ventral to the skull, and for the ACC: 1.2 mm anterior to bregma, 0.8 mm lateral to the midline, and 2 mm ventral to the skull (Supplementary Figure 2). Wires were joined to a 5-pin mini-Molex plug (13.5×3×7 mm, 0.2 g), and fixed to the skull by acrylic cement (Vertex, MA, USA; n = 8 rat). 

### Neural recording and wavelet analysis 

The Continuous Wavelet Transform (CWT) is applied to show a time-series signal in a time-frequency framework. A translated version of the genuine wavelet function ψ0 and the criterion time-series signal can be shown as a twist output. The genuine wavelet function has various criterions that are stretched and condensed in time. It is needed that the genuine wavelet be translated different times along the time axis to show all duration of the signal because its contract version cannot show the whole duration of the time-series signal. The conversion of the contiguous wavelet with a time series x(n), (n=1,2,3,…, N), which is sampled from a contiguous sample at a time stage Δt is explained below:


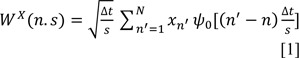


Here n represents the translation factor and s is the criterion factor, which is inversely proportional to the frequency. The similarity between the translation/criterion versions of the real wavelet and the signal is described by the CMT ratio.

The connectivity of the wavelet which assesses the degree of cohesion between two time series signals in separate frequency and time ranges can be determined conforming to the CWT. The cohesion of the squared cross-wavelet is following:


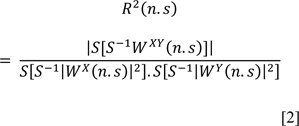


W^XY^ = W^X^W^Y*^ is a cross wavelet-transformation of two-time series x(n) and y(n) and * denotes the complex conjugate. 

Here S applied to determine a good measure of correlation as a smoothing operator.

The correlation between two signals (time series) is demonstrated by R^2 (n.s) and it is confined between 0 and 1. The coherency of two time series signals in a separate time and frequency scales can be the explanation of the squared cross-wavelet. The analytical morlet wavelet is considered a suitable way to calculate the time-frequency coherence detection because it provides an appropriate balance between frequency and time localization (Grinsted et al., 2004[14]).

The correlation of the wavelet is counted outside the cone of influence (COI) and because the areas inside the COI are affected by the edge effects then areas inside COI likely are not efficient. To get the significance level for the correlation, R^2 (n.s) the value of the surrogate data was compared with the correlation of the actual data (Maraun and Kurths, 2004[27]). Bootstrapping was applied for creating a large (n=1000) number of deputy data pairs and then for each of those data pairs the correlation was assessed. P < 0.05 was measured for the significance of the compared simulated and actual data pairs. A mercantile acquisition processor (Niktek, http://niktek.ir/) was applied to record, filter and digitalize the neural activities for aspirating LFPs (at the sampling rate of 1000Hz). Analysis of wavelet correlation was done by wavelet toolbox of Matlab software version 2018a (The MathWorks, Inc., US).

### Wavelet analysis

A wavelet is a wave, e.g. an oscillation, which has a limited duration and whose average is zero:


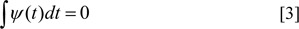


Based on *ψ*(*t*) which is named “mother wavelet”, other functions can be produced:


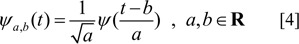


The permanents *a* and *b* are named scale and translation, respectively. The functions produced by parity (2) are the same functions as parity (1) which are translated and dilated/ condensed. 

The consecutive wavelet transform (CWT) of a time-series function x[*n*] is described as:


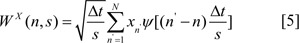


For a permanent translation *n* and scale *s*, the CWT multiplier shows the likeness between the function x[*n*] and *ψ*(*n,s*). The higher the similarity, the grater the CWT multipliers and vice versa. Using CWT, it is conceivable to evaluate the degree of correlation in various scales and time points between two functions. The squared cross-wavelet correlation is determined as:


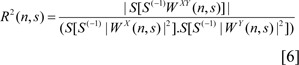


In equation [6] *s* is a smoothing operator applied to give a beneficial evaluation of correlation. *R*^2^(*n,s*) evaluates the correlation between two time-series signals. It can be translated as the coherence between two signals in particular time and scale ranges. The amount of squared cross-wavelet correlation ranges between 0 and 1; 0 means lack of correlation and 1 means complete correlation.

Recordings, digitalization and filtering of neural activities were done using a mercantile acquisition processor (Niktek, http://niktek.ir/). Recordings were bandpass-filtered (0.01-250 Hz) to aspirate LFPs and then sampled at 1000 Hz. Wavelet correlation analysis was done using the wavelet toolbox of Matlab software 2018a (The MathWorks, Inc., US). Time-frequency correlation representations were computed using the analytic morlet wavelet that is a good choice since it prepares a excellence parity between time and frequency localization (Grinsted et al., 2004[14]). The magnitude of correlation of actual data was compared with the correlation of deputy data to receive the remarkable level of correlation, *R*^2^(*n,s*). In particular, a large number (n=1000) of deputy data pairs were constructed using bootstrapping and then the correlation was assessed for each of these data pairs. The correlation of the actual data pair was compared with all of these simulated data pairs and was measured significant in areas where p<0.05.

### Experimental design

After recovery of surgery, rats (n = 8) were retrained to the maze until preferring great reward choice over 80 % for three following days to sure they prefer great reward choice as before surgery. Then, before starting the morphine dependence procedure (last session before the first morphine treatment day), rats performed ten choice trials, and percentage of great reward/great effort arm, and/or low reward/low effort arm choices was investigated for each rat, and local field potential recording (LFP) was recorded from the ACC and BLA simultaneously during each trial. 

After that, the animals received morphine (twice per day for 10 days) for induction of morphine dependence. Each day, three hours after the morphine injection (to ensure that decision making was not related to acute morphine treatment but to the previous morphine exposure), the animals performed ten decision trials. On test day, 10^th^ day of morphine dependence procedure, rats were placed on the T-maze and the performed ten choice trials. Percentage of choice of great reward/great effort arm, and/or low reward/low effort arm was assessed for each rat, and LFP was recorded from the ACC and BLA simultaneously during each trial. After behavioral and electrophysiological tests, the animals received naloxone (2 mg/kg), and behavioral manifestations of withdrawal according to a modified version of the Gellert-Holtzman scale (Gellert and Holtzman, 1978), were assessed (Supplementary Figure 1B). 

The locomotor activity of the rats before morphine dependence and during the period of morphine dependence was recorded to ensure that the effects of morphine dependence on decision making were not due to hypoactivity or hyperactivity. The animals were located in the center of an open field measuring 30 cm × 30 cm with 30 cm walls and were permitted to freely move inside the arena for 30 min. A 3CCD camera (Panasonic Inc., Japan) mounted 2 m above the open field, and recorded the distanced travel and locomotor activity. Data was analyzed by offline sing Ethovision video tracking software (version 3.1, Noldus Information Technology, The Netherlands).

Besides, to investigate possible involvement of getting older or laboratory condition in choose of low reward arm in morphine-dependent animals, a further naive-control group (n = 8) performed decision-making task, but received subcutaneous injection of saline (instead of morphine). Percentage of choice of great reward/great effort, and/or low reward/low effort was evaluated for each rat in this group (Supplementary Figure 3A).

### Statistics

The behavioral data was analyzed by commercially available software GraphPad Prism® 5.0. T-test and/or repeated measures one-way ANOVA followed by post hoc Dunnett multiple comparison tests were used. *P*-values less than 0.05 (*P* < 0.05) were measured to be statistically significant.

## Results

### Effects of morphine dependence on the great reward choice percentage and movement activity in an effort-based decision-making task

**Figure 1:**
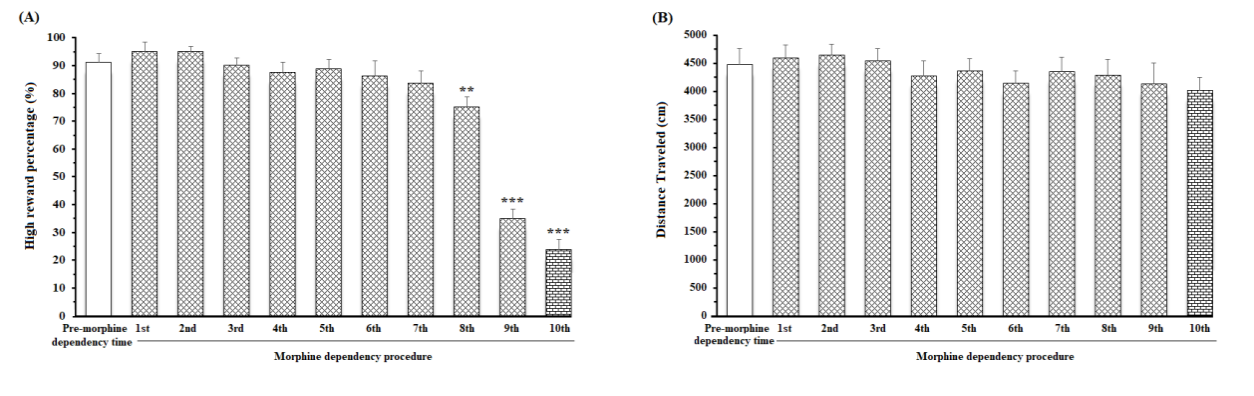
(A) Morphine dependence decreased the percentage of great reward/great effort choice in the effort-based T-maze decision making task. (B) Morphine dependence did not have any significant effect on locomotor activity. Data are shown as mean ± SEM for 8 rats. *** *P* < 0.001, ** P < 0. 01 different from the pre-morphine dependence time

The animals performed effort-based T-maze decision-making tasks (great reward/great effort vs. low reward/low effort), repeated measures one-way ANOVA followed by post hoc Dunnett multiple comparison tests [F=56.12, P<0.0001] revealed that morphine dependence (10^th^ day of morphine dependence procedure) decreased the percentage of great reward choice compared to pre-morphine dependence time (last session before the first morphine treatment day). In fact, the morphine dependence changed the preference of animals to select a low reward/ low effort instead of a great reward/great effort (Figure 1A(Fig. 1)). 

There is also a risk that the changes in decision-making after morphine dependence are due to the change in locomotor activity, so that morphine dependence could lead to hypoactivity or hyperactivity. To evaluate this potentially confounding variable, in an effort-based decision-making task, the animals were located in an open field in both pre-morphine dependence and morphine dependence times, after the decision-making test and were permitted to explore for 30 min. Movement activity and distanced travel were assessed. A one-way ANOVA with repeated measures followed by Dunnett's comparison tests [F=0.5945, P=0.8131] showed that morphine dependence had no significant effect on the distance traveled compared to the time before morphine dependence (Figure 1B(Fig. 1)).

### ACC-BLA coherence during greatreward/great effort choice in effort-based decision-making task in pre-morphine dependence time

**Figure 2:**
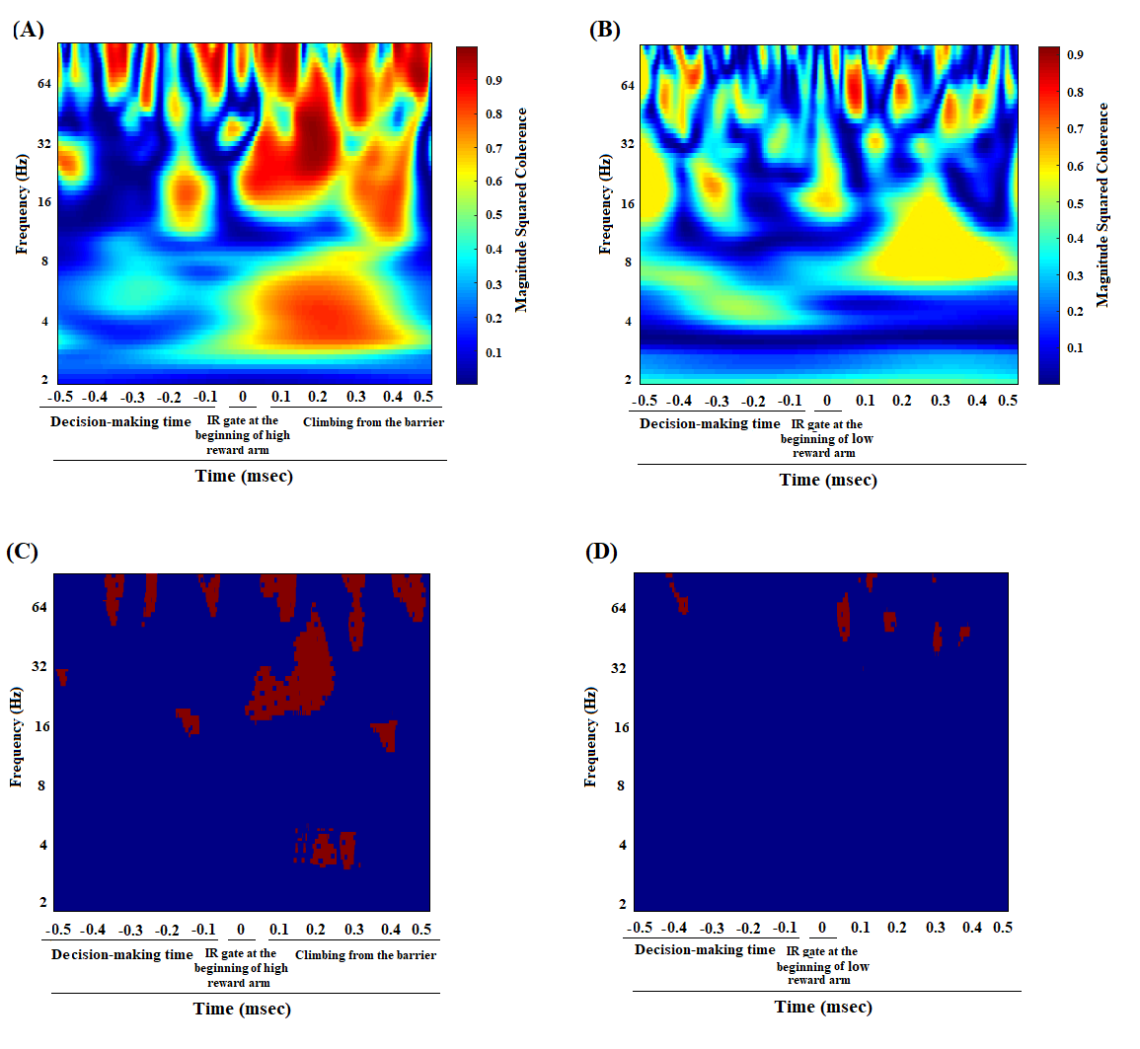
Comparison of coherence between great reward/great effort choice trials in pre-morphine dependence time (last session before the first morphine treatment day) (n=51 trials from 8 rats) (A), and low reward/low effort choice trials in morphine dependence time (10^th^ day of morphine dependence procedure) (n=48 trials from 8 rats) (B) during effort-choice decision-making. Time-frequency representation of group averages coherence between the ACC and BLA (500 ms before and 500 ms after breaking the infrared sensor at the beginning of the goal arm). The cone of influence (COI) where edge effects should be considered is blurred. It should be noted that the wavelet scales are converted to approximate frequencies on the graph. Comparison of areas in which the coherence is significantly different compared to a large number of surrogate data between great reward/great effort choice trials in pre-morphine dependence time (C), and low reward/low effort choice trials in morphine dependence time (D). The red regions in figures show the areas with significant difference (P < 0.05). Surrogate data were simulated using bootstrapping (n = 1000).

**Figure 3:**
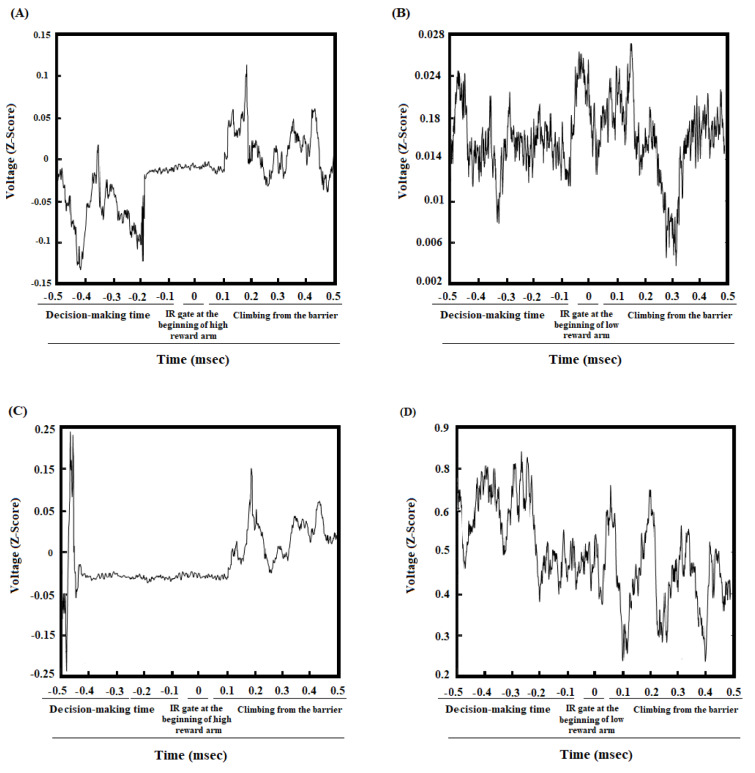
Z-normalized time-domain signals of the ACC. The group's time-domain averages signal acquired from the ACC in great reward/great effort choice trials in pre-morphine dependence (last session before the first morphine treatment day) (A), and low reward/low effort choice trials in morphine dependence (10^th^ day of morphine dependence procedure) (B). Z-normalized time range of the group average signals recorded by the BLA. The group's time-domain averages signal acquired from the BLA in great reward/great effort choice trials in pre-morphine dependence time (C), and low reward/low effort choice trials in morphine dependence time (D).

The coherence between the ACC and BLA of group average of great reward/great effort choice trials in pre-morphine dependence time was shown in Figure 2A(Fig. 2). During making an effort-based choice, a significant enhancement in gamma oscillation, and a poor enhancement in beta oscillation were observed. After poking the IR gate at the beginning of great reward goal arm and during expending effort and climbing the barrier, there was a significant increase in delta, theta, beta, and gamma frequency bands. Figure 2C(Fig. 2) revealed that the regions in which the correlation is significant compared to successor data (n = 1000, *P* < 0.05) by using bootstrapping (Figure 2A and C(Fig. 2)). The alteration of frequency range can also be considered by looking at the group average z-normalized time-domain signals of ACC and BLA regions in pre-morphine dependence time (Figure 3A and C(Fig. 3)). 

### ACC-BLA coherence during low reward/low effort choice in effort-choice decision-making task in morphine dependence time

Based on Figure 2B(Fig. 2), it appears that during making a decision for preference of low reward/low effort in morphine-dependent animals, a weak coherence between the ACC and BLA areas in gamma frequency bands was observed which is not considerable. These animals chose to not climb a barrier and expend low effort. As a result, in contrast to preference of great reward/great effort in pre-morphine time, no increase in delta, theta, and beta frequencies was observed during expending effort in morphine-dependent animals. However, there was an increase in gamma during expending effort but it was weaker compared to great reward/great effort choices during pre-morphine dependence. The regions in which correlation is remarkably shown in Figure 2D(Fig. 2) that, again bootstrapping (n = 1000, P < 0.05) has been used to recognize significance. Group average z-normalized time-domain signals of ACC and BLA areas in morphine dependence time showed that the change in signal's amplitude of ACC (Figure 3B(Fig. 3)) and BLA (Figure 3D(Fig. 3)) areas was different from pre-morphine dependence time.

**Figure 4:**
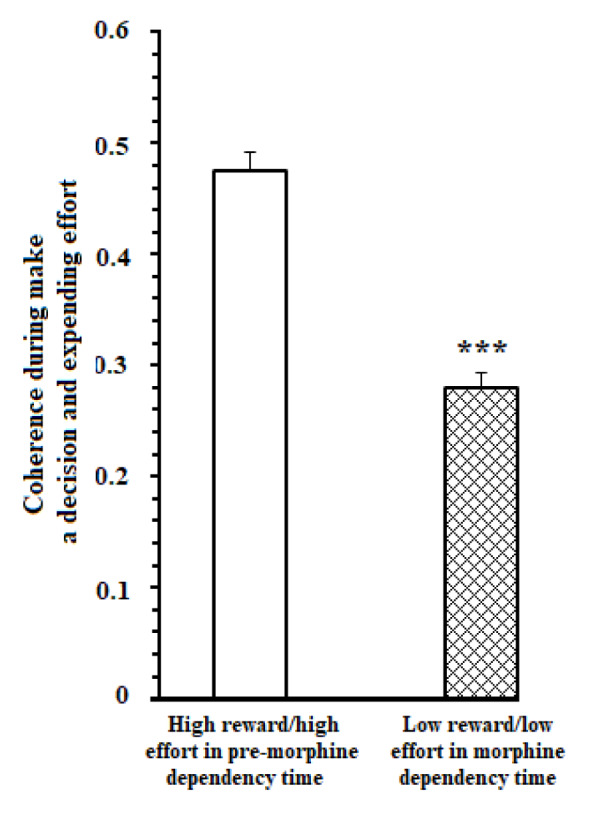
Mean ± SD of ACC-BLA coherence during making a decision and expending reward in 1000 ms time window (500 ms before and 500 ms after breaking the infrared sensor at the beginning of the goal arm). *** *P* < 0.001

Besides, a comparison of coherence values in great reward/high effort trials in pre-morphine dependence time and low reward/low effort trials in morphine dependence time during the 1000 ms time window was performed. Results [t=9.183, P<0.0001] showed that in reward reward/high effort trials in pre-morphine dependence time, functional activity in delta, theta, beta, and gamma frequencies remarkably was higher than those of low reward/low effort trials in morphine dependence times (Figure 4(Fig. 4)).

In addition, the results of the comparison of reward reward/great effort choice between naïve-control group and morphine-dependent group showed that the preference of low reward/low effort in morphine-dependent animals was not related to getting older or laboratory condition [t=16.25, P<0.0001] (Supplementary Figure 3A).

### ACC-BLA coherence in great reward/great effort choice while reaching the reward in pre-morphine dependence time 

**Figure 5:**
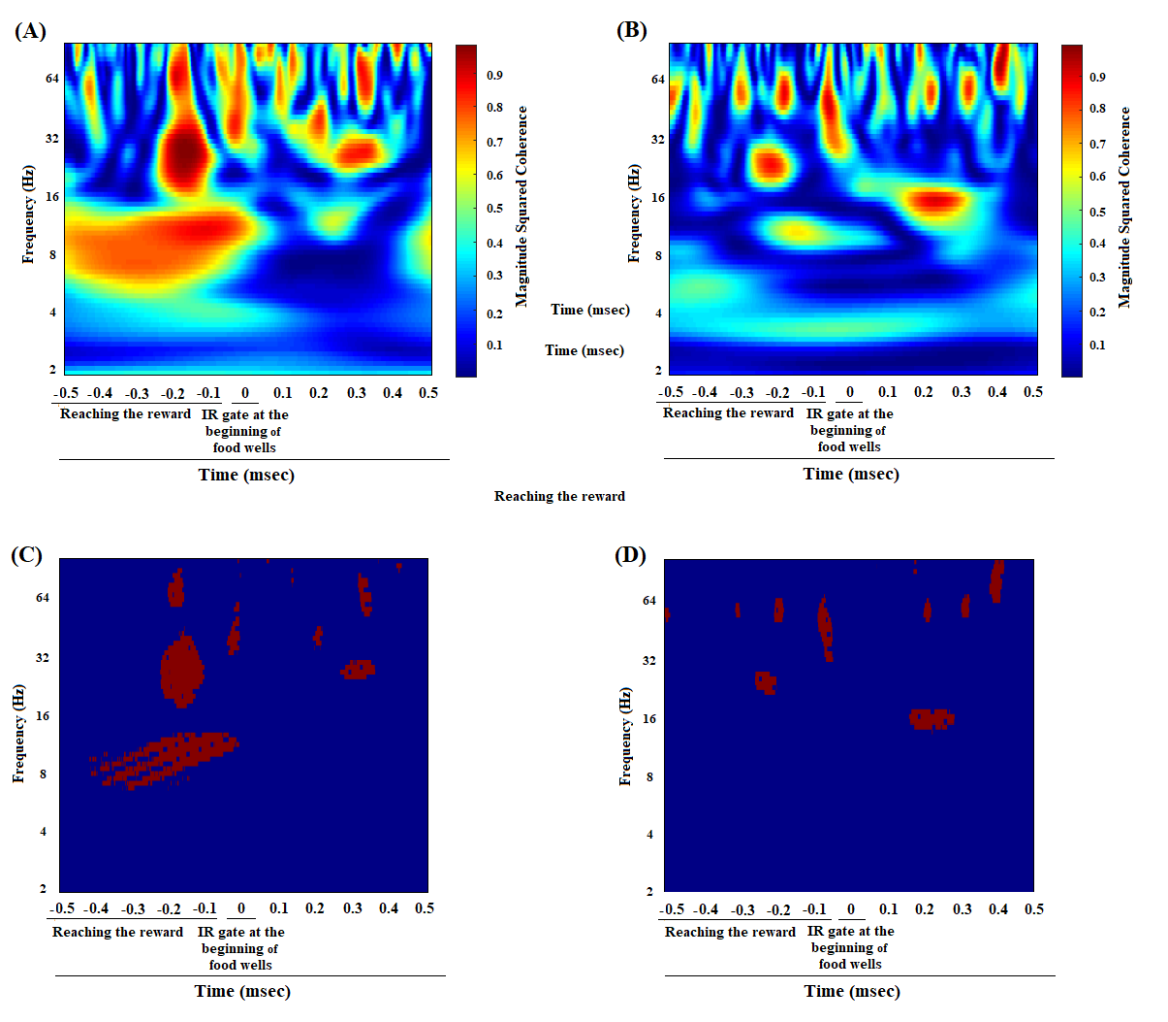
Comparison of coherence between great reward/great effort choice trials in pre-morphine dependence time (last session before the first morphine treatment day) (n=51 trials from 8 rats) (A), low reward/low effort choice trials in morphine dependence time (10^th^ day of morphine dependence procedure) (n=48 trials from 8 rats) (B) while reaching to reward. Time-frequency representation of group averages coherence between the ACC and BLA (500 ms before and 500 ms after breaking the infrared sensor at the beginning of the food well). The cone of influence (COI) where edge effects should be considered is blurred. It should be noted that the wavelet scales are converted to approximate frequencies on the graphs. Comparison of areas in which the coherence is significantly different compared to a large number of surrogate data between great reward/great effort choice trials in pre-morphine dependence time (C), low reward/low effort choice trials in morphine dependence time (D) while reaching a reward. The red regions in figures show the areas with significant difference (P < 0.05). Surrogate data were simulated using bootstrapping (n = 1000).

**Figure 6:**
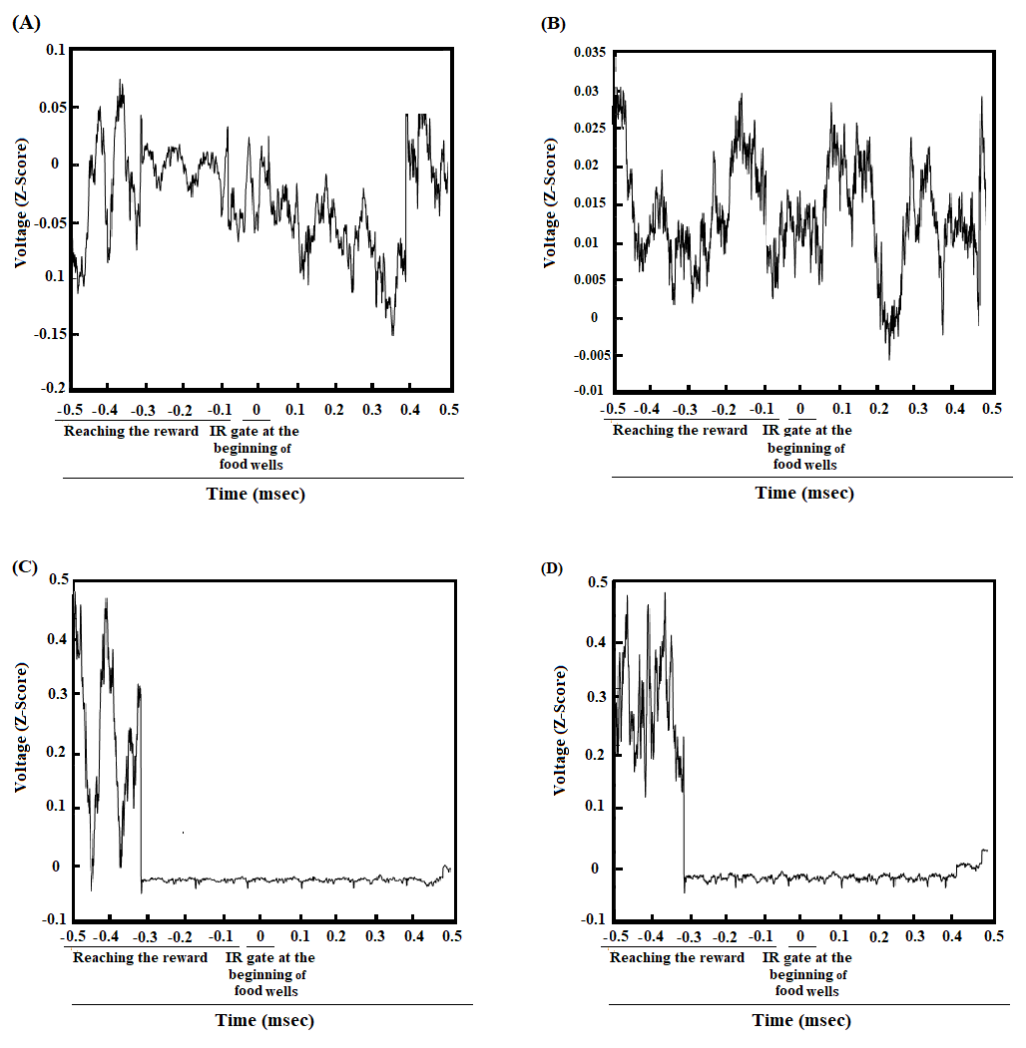
Z-normalized time-domain signals of the ACC. The group's time-domain averages signal acquired from the ACC in great reward/great effort choice trials in pre-morphine dependence time (last session before the first morphine treatment day) *(A)*, and low reward/low effort choice trials in morphine dependence time (10^th^ day of morphine dependence procedure) *(B)* while reaching a reward (500 ms before and 500 ms after breaking the infrared sensor at the beginning of the food well). Z-normalized time-domain of group average signals acquired from the BLA. The group's time-domain averages signal acquired from the BLA in great reward/great effort choice trials in pre-morphine dependence time *(C)*, and low reward/low effort choice trials in morphine dependence time *(D)* while reaching a reward.

Figure 5A(Fig. 5) revealed the correlation between the ACC and BLA of group average of reward reward/great effort choice trials in pre-morphine dependence time while reaching the reward. A strong enhancement in theta, beta, and gamma oscillations has occurred before breaking infrared sensor at the beginning of food well and during reaching the reward. Figure 5C(Fig. 5) clarified that the regions in which the correlation is significantly compared to successor data (n = 1000, P < 0.05) using bootstrapping. Group average of the z-normalized time-domain signals of the ACC and BLA areas, showing the signal amplitude in the ACC and BLA in the time before morphine dependence, are shown in Figure 6A and C(Fig. 6), respectively.

### ACC-BLA coherence in low reward/low effort choice while reaching the reward in morphine dependence time 

Based on Figure 5B(Fig. 5), the coherence between the ACC and BLA of the group average of the low reward/low effort choice trials in the morphine dependence trials (before the infrared sensor breaks at the beginning of the food well and the reward is reached), was in the beta and gamma frequencies. However, this increase in beta and gamma frequencies was smaller compared to pre-morphine dependence period. The regions in which correlation is remarkably shown in Figure 5D(Fig. 5) that, again bootstrapping (n = 1000, *P* < 0.05) has been used to recognize significance. Figure 6B and 6D(Fig. 6) show signal's amplitude of the ACC and BLA I morphine dependence time. 

**Figure 7:**
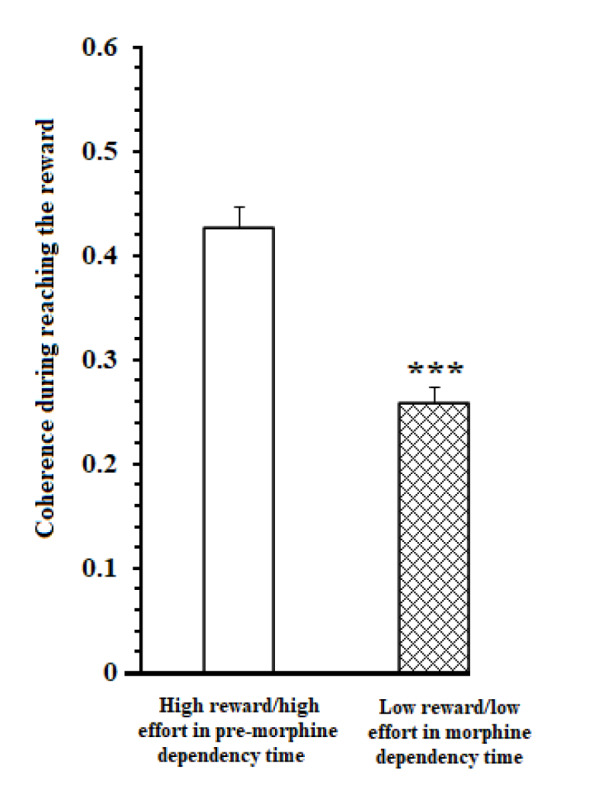
Mean ± SD of ACC-BLA coherence during reaching reward in 1000 ms time window (500 ms before and 500 ms after breaking the beam the infrared sensor at the beginning of the food well). *** *P* < 0.001

On the other hand, a comparison of coherence values in reward reward/high effort trials in pre-morphine dependence time and low reward/low effort trials in morphine dependence trials during the 1000 ms time window while reaching to reward was performed. Result [t=6.325, *P* =0.0001] revealed that in reward reward/high effort trials, functional activity in theta, beta, and gamma frequencies remarkably was higher than those of low reward/low effort trials in morphine dependence times (Figure 7(Fig. 7)). 

See also the Supplememtary data.

## Discussion

This study investigated the effects of morphine dependence on the ACC-BLA neural synchronization during making an effort-choice decision and expending effort. Results revealed ***(I)*** in pre-morphine dependence time, neural coherence between the ACC and BLA was observed in beta and gamma frequencies during making a great reward/great effort choice, and in delta, theta, beta, and gamma frequencies during expending effort and climbing the barrier. ***(II)*** In morphine dependence time, there was not any significant coherence in beta frequency during making a low reward/low effort choice, however, there was a weak coherence in gamma frequency. The animals chose to expend low effort and did not climb the barrier, and there was not any coherence between delta, theta, and beta, however, a coherence in gamma was observed. ***(III)*** During reaching the great reward in pre-morphine dependence time, a high degree of coherence was shown in theta, beta, and gamma frequencies between the ACC and BLA. However, during reaching the low reward in morphine dependence time, this coherence became weaker compared to reaching great reward in pre-morphine dependence time.

In the pre-morphine addiction period, when the animals broke the IR gate at the end of the initial arm and heard a tone to illuminate the large reward arm, ACC-BLA coherence performance in beta and gamma increased significantly when they chose the large reward/large effort arm (Figure 2A and C(Fig. 2)). However, if they chose low reward/low effort arm, the increase in beta was not significant, and the increase in gamma was weaker compared to great reward/great effort choice (Supplementary Figure 3B, Panel A). Besides, when the animals chose great reward/great effort arm, they have to expend effort and climb the barrier which was accompanied by the enhancement in delta, theta, beta, and gamma (Figure 2A and C(Fig. 2)). However, when animals preferred low reward/low effort, they did not need much effort and did not have to climb the barrier in which case, the increase in delta, theta, and beta frequency bands was not observed, and there is a weak coherence in gamma oscillation (Supplementary Figure 3B, Panel A). On the other hand, when the animals were reaching great reward, a significant increase in theta, beta, and gamma was observed (Figure 5 A and C(Fig. 5)). 

In morphine-dependent rats, when the animals made a low reward/low effort choice, a weak increase in gamma frequency between the ACC and BLA was observed, however, there was not any significant increased coherence in beta oscillation (Figure 2B and D(Fig. 2)). Nonetheless, when they made great reward/great effort choice, there was a significant increase in beta/gamma oscillations (Supplementary Figure 3B, Panel B). Besides, during reaching the low reward, it was observed the ACC-BLA synchronization in beta and gamma, however, it was weaker compared to reaching the great reward in great reward/great effort choices. 

In previous studies, intracranial electrophysiology and functional neuroimaging reveal that the ACC and amygdala are implicated in assessing values and remunerations to guide future behavior (Basten et al., 2010[2]; Jenison et al., 2011[19]) and generate alteration of choice associated with the ACC and amygdala activity (Sokol-Hessner et al., 2013[37]). Functional imaging studies have reported the amygdala activation when choosing options related to greater reward magnitudes (Hsu et al., 2005[18]; Martino et al., 2006[28]). The BLA received various sensory information both from the frontal cortex and thalamus, sending much projection to cortical and subcortical regions involving the hippocampus (McDonald, 1998[29]; Pitkanen et al., 2000[35]), striatum, orbitofrontal cortex (Kita and Kitai, 1990[23]), and ACC. Pharmacological inactivation of the BLA altered rats' predilection from high-reward options to low-reward in effort or delay-based decision-making tasks (Winstanley et al., 2004[40]). Acute doses of morphine led to an enhancement in distinction for smaller immediate rewards rats (Harvey-Lewis and Franklin, 2015[16]). Chronic morphine injection has long-lasting effects on choice behavior (Eppolito et al., 2013[4]), such that chronic exposure to morphine impaired effort- or delay-based forms of cost-benefit decision-making (Fatahi et al., 2020[7][9]). It was also found that those who used heroin exhibited more significant delay discounting (Karakula et al., 2016[20]), and heroin users displayed remarkably more detrimental decision-making and longer contemplation times during making risky decisions than the control groups (Li et al., 2013[26]; Zhang et al., 2012[41]). Individuals using heroin show a significantly more preference for immediate rewards than healthy comparison samples (Eppolito et al., 2013[4]; Karakula et al., 2016[20]). They also suffer from a lack of sensitivity to future consequences, difficulties in regulating their behavior and impulsiveness (Morein-Zamir and Robbins, 2015[31]; Verdejo-Garcia et al., 2008[39]). 

In the current study, the animals preferred the "great reward/great effort" arm in almost 90 % of cases in the period before morphine withdrawal, i.e. they preferred to receive a great reward when they had to exert themselves. In these trials, the power of beta and gamma frequencies was increased when the animals make an effort-based decision, and also power of delta, theta, beta, and gamma oscillations had a strong increase while the animals expend effort to climb the barrier. However, morphine dependence led the animals to prefer the low reward, low effort arm in almost 75 % of trials, so morphine-dependent animals preferred low reward with low effort. In these trials, the enhancement of beta was not observed, and the increase of gamma was weaker compared to great reward/great effort trials. Therefore, it can be concluded that ACC-BLA coherence and increase in beta/gamma oscillations is essential for making an effort-based decision, and also expending effort for getting great reward is associated with the increase of delta, theta, beta, and gamma frequencies. It is possible that the impairment of effort decisions and effort in morphine-dependent animals is related to disturbance of neural synchronization between ACC and BLA.

On the other hand, during reaching the reward, there was neural coherence between the ACC and BLA and power of theta, beta, and gamma frequency bands increased. However, this increase in great reward/great effort choices was more remarkable compared to low reward/low effort choices. It may be possible that the coherence of theta, beta, and gamma frequencies between the ACC and BLA has a role in the motivation to reach higher reward.

Overall, this study has shown that neural coherence and functional connectivity between the ACC and BLA plays a fundamental role in beta and gamma frequencies in effort-based decision-making, in delta, theta, beta, and gamma frequencies in effort, and in theta, beta, and gamma oscillations in reward attainment. Effects of morphine dependence on the preference of low reward/low effort, expending low effort, and tendency to low reward, is partly related to the impairment of neural synchronization between the ACC and BLA.

## Declaration

### Acknowledgments

This project was supported by grant (No. 99007925) from the Iran National Science Foundation, Tehran, Iran. The authors also would like to thank the Neuroscience Research Center, Shahid Beheshti University of Medical Sciences, for cooperating with this study.

### Ethical statement

All experiments were done in accordance with the National Institutes of Health Guide for the Care and Use of Laboratory Animals (NIH Publication; 8th edition, revised 2011) and were approved by the Research and Ethics Committee of School of Medicine, Shahid Beheshti University of Medical Sciences (IR.SBMU.PHNS.REC. 1398.136), Tehran, Iran.

### Author contributions

Zahra Fatahi: Experiment design, implementing the experimental task and collecting data, analysis and interpretation of results and writing the manuscript; Mohammad Fatahi: Analysis and interpretation of results; Abbas Haghparast: Experiment design, supervision, resources, funding acquisition.

### Conflict of interest

The authors declare no conflict of interest.

## Supplementary Material

Supplementary information

Supplementary data ACC-10th day of morphine dependence

Supplementary data ACC-Premorphine injection

Supplementary data AMY-Premorphine injection

Supplementary data AMY-10th day of morphine dependence
