# Supplementary material for: Impairment of value-based decision-making in morphine-dependent rats is partly related to neural connectivity between the anterior cingulate cortex and basolateral amygdala
Source: EXCLI J. 2024 Jan 3;23:1–17. doi: 10.17179/excli2023-6442 (PMC10853632; doi:10.17179/excli2023-6442)
Supplement: Supplementary information [file EXCLI-23-1-s-001.pdf]

EXCLI Journal 2024;23:1-17 – ISSN 1611-2156
Received: August 05, 2023, accepted: November 30, 2023, published: January 03, 2024

Supplementary information to:

Original article:

IMPAIRMENT OF VALUE-BASED DECISION-MAKING IN MORPHINE-DEPENDENT RATS IS PARTLY RELATED TO NEURAL CONNECTIVITY BETWEEN THE ANTERIOR CINGULATE CORTEX AND BASOLATERAL AMYGDALA

Zahra Fatahi[a], Mohammad Fatahi[b], Abbas Haghparast[a,c,d,*]

[a] Neuroscience Research Center, Shahid Beheshti University of Medical Sciences, School of Medicine, Tehran, Iran
[b] School of Electrical and Computer Engineering, College of Engineering, University of Tehran, Tehran 1439957131 Iran
[c] School of Cognitive Sciences, Institute for Research in Fundamental Sciences, Tehran, Iran
[d] Department of Basic Sciences, Iranian Academy of Medical Sciences, Tehran, Iran

* **Corresponding author:** Abbas Haghparast, PhD, Neuroscience Research Center, Shahid Beheshti University of Medical Sciences, P.O.Box 19615-1178, Tehran, Iran; Tel/fax: +98-21-2243-1624; E-mail: Haghparast@yahoo.com; Haghparast@sbmu.ac.ir

https://dx.doi.org/10.17179/excli2023-6442

This is an Open Access article distributed under the terms of the Creative Commons Attribution License (http://creativecommons.org/licenses/by/4.0/).

EXCLI Journal 2024;23:1-17 – ISSN 1611-2156
Received: August 05, 2023, accepted: November 30, 2023, published: January 03, 2024

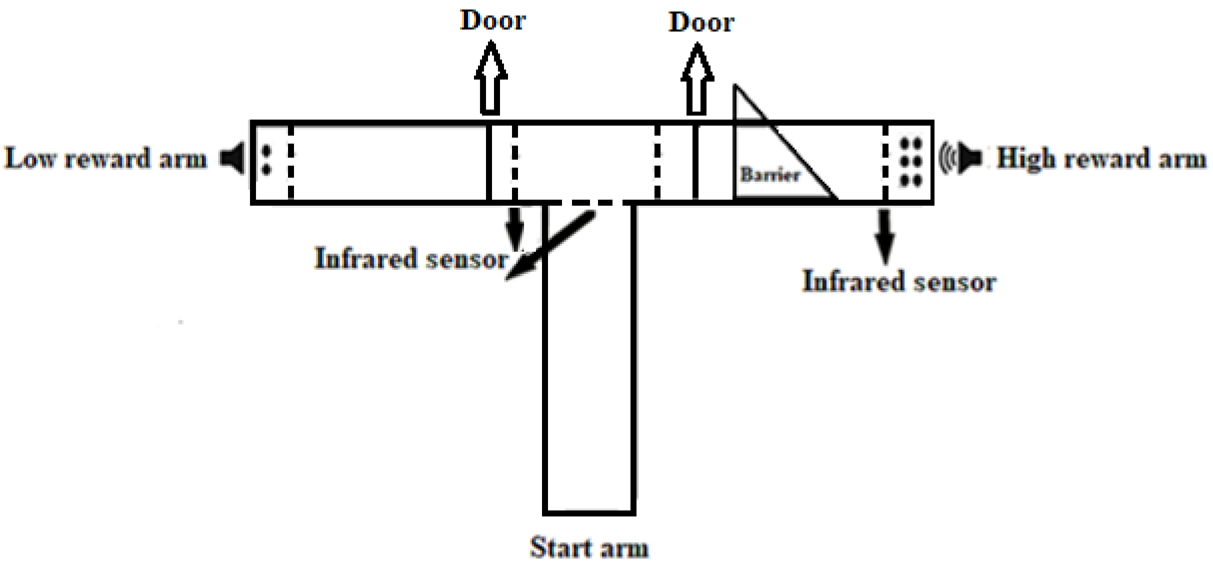

Supplementary Figure 1A: Schematic illustration of the apparatus. Effort-based T-maze decision-making task. The apparatus has three arms including start, high reward and low reward arms. Rats could choose to climb a barrier (30 cm) in order to obtain a higher reward (6 pieces of reward), and/or choose to receive a small reward (2 pieces of reward) without physical effort. Infrared sensors were placed at the end of the start arm, at the beginning of the goal arms, and at the beginning of food wells.

As soon as the animals have broken through the infrared sensor at the end of the start arm, a sound signal sounds from a loudspeaker at the end of the high reward arm to determine the high reward arm for the animals. The animals then choose the high reward arm and/or the low reward arm. When the animals enter the target arm, they break through the infrared sensor at the beginning of the target arm. The time between the breaching of these two infrared sensors (at the end of the start arm and the beginning of the target arm) was approximately 500 ms, and the recording of the local field potential at this time was analyzed as neural activity during the decision time. In addition, 500 ms after the breaching of the infrared sensor at the beginning of the target arm was analyzed to assess neural coherence during the effort and climbing of the barrier.

EXCLI Journal 2024;23:1-17 – ISSN 1611-2156
Received: August 05, 2023, accepted: November 30, 2023, published: January 03, 2024

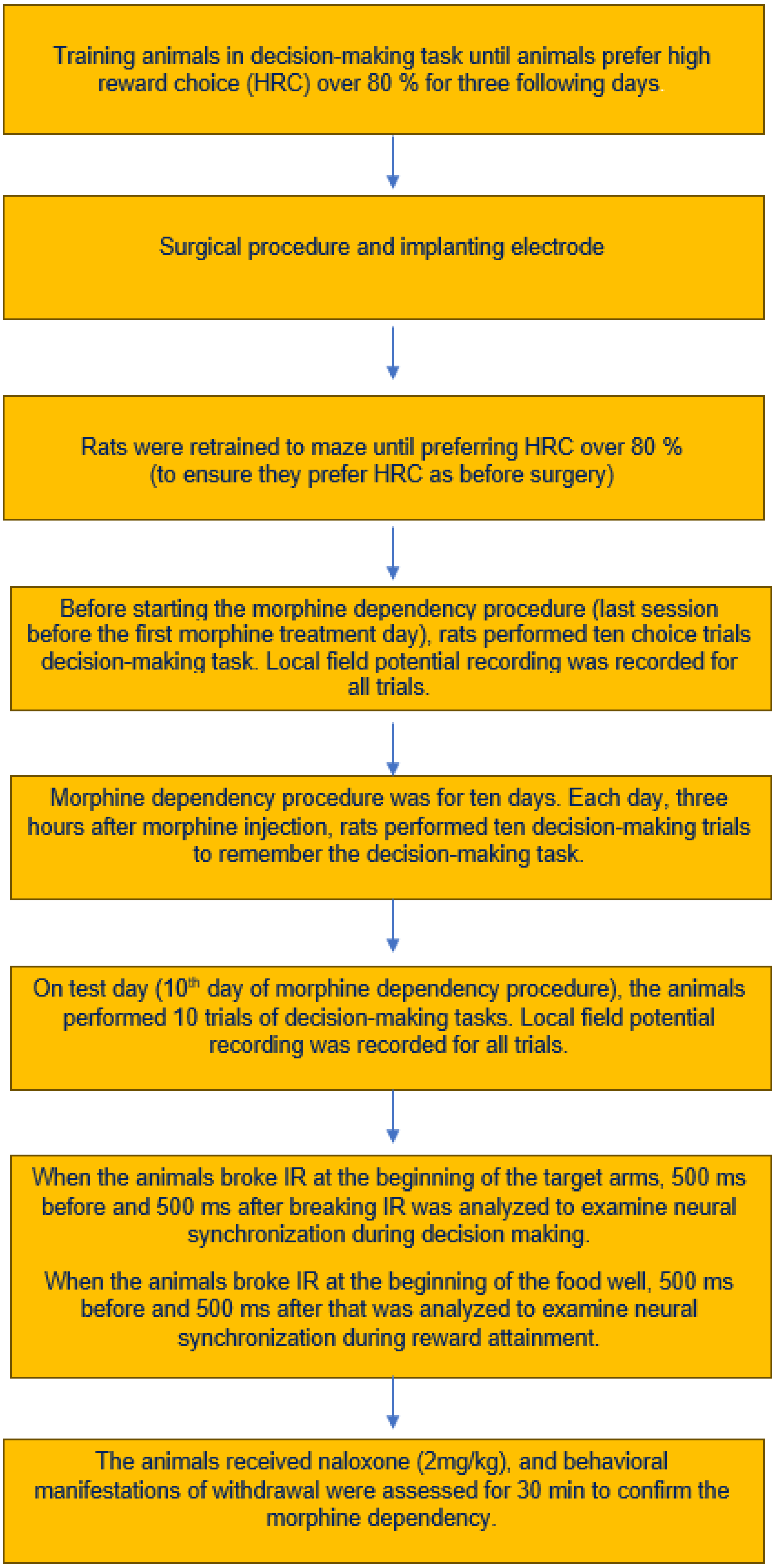

Supplementary Figure 1B: Schematic paradigm of experimental design

EXCLI Journal 2024;23:1-17 – ISSN 1611-2156
Received: August 05, 2023, accepted: November 30, 2023, published: January 03, 2024

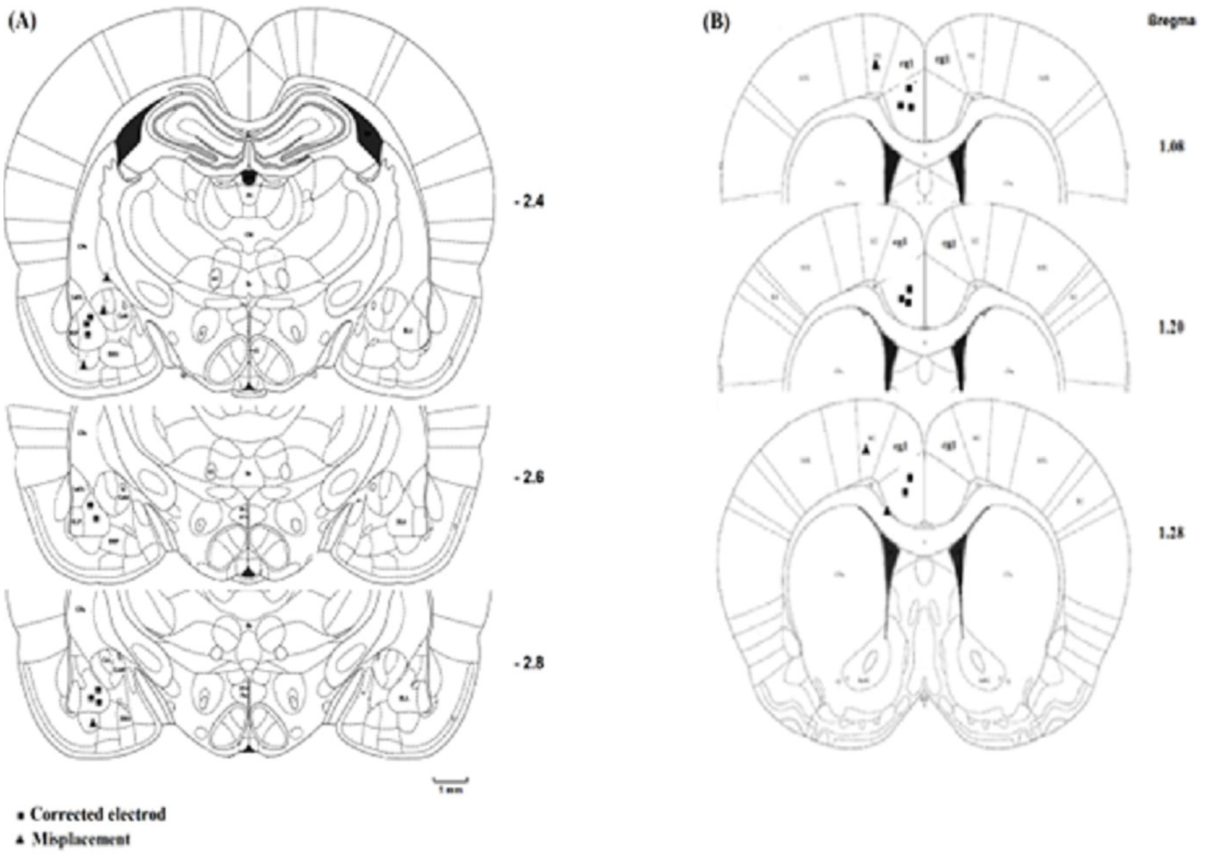

Supplementary Figure 2: Anatomical reconstruction of electrode tips in the BLA **(A)** and ACC **(B)**. Corrected electrode tips (n = 8 rat), misplacement

EXCLI Journal 2024;23:1-17 – ISSN 1611-2156
Received: August 05, 2023, accepted: November 30, 2023, published: January 03, 2024

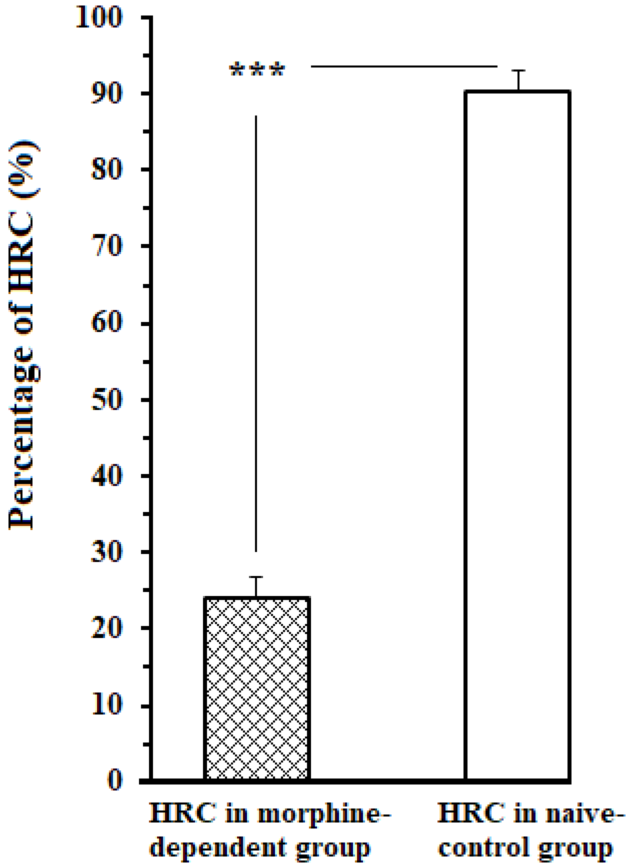

Supplementary Figure 3A: Another naïve-control group (n = 8) performed the decision task but received subcutaneous injection of saline (instead of morphine) to test whether aging and/or laboratory conditions play a role in the choice of the low reward arm in morphine-dependent animals. The percentage of choice of high reward (HRC) and/or low reward/low effort was recorded for each rat in this group. The results showed that the preference for low reward/low effort in morphine-dependent animals was not related to age or laboratory conditions.

EXCLI Journal 2024;23:1-17 – ISSN 1611-2156
Received: August 05, 2023, accepted: November 30, 2023, published: January 03, 2024

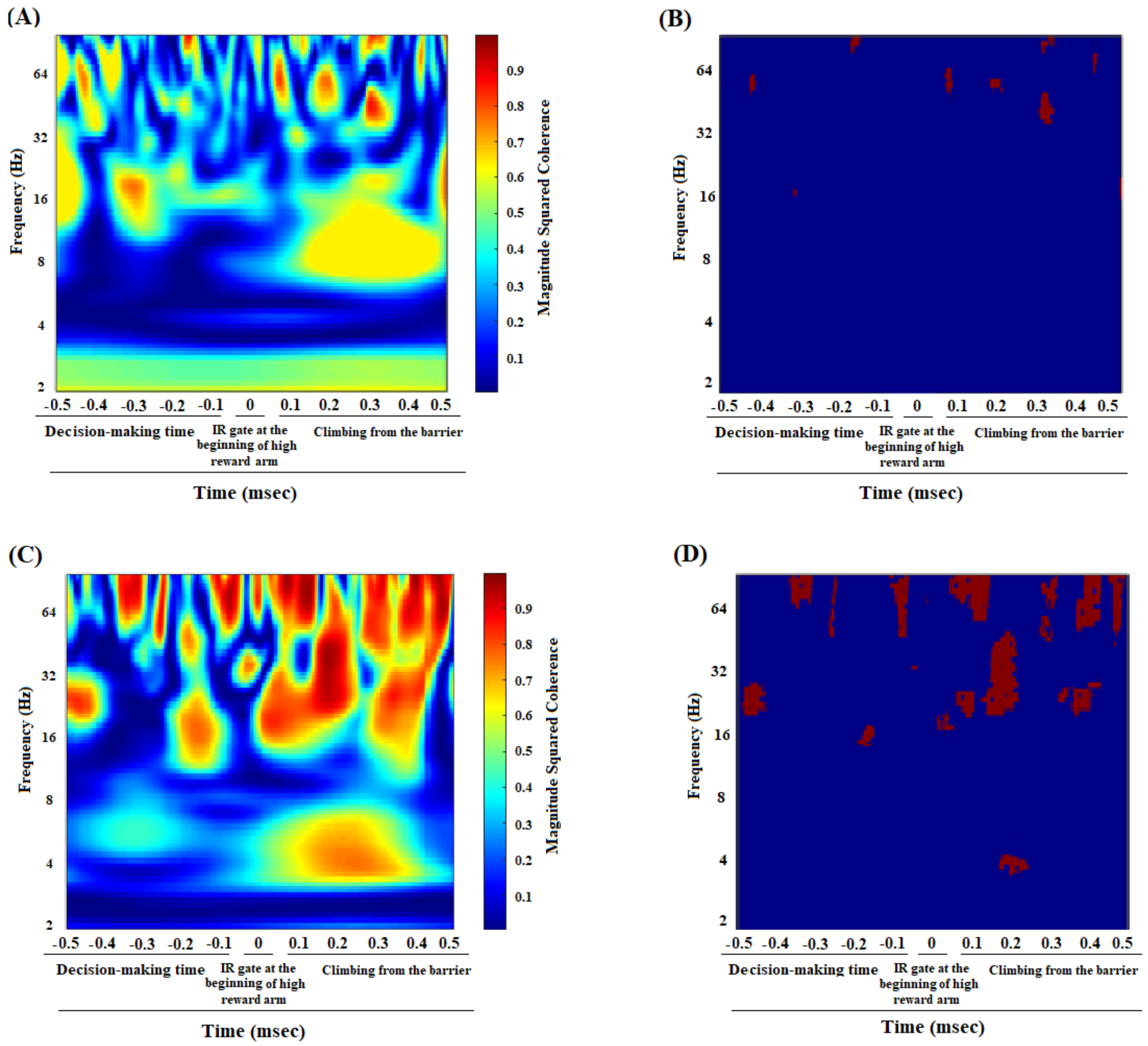

Supplementary Figure 3B: Comparison of group mean coherence between low reward/low effort trials during decision making in pre-morphine addiction period **(A and B)**, and high reward/high effort trials during decision making in morphine addiction period **(C and D)**. Time-frequency plot of group average coherence between the ACC and AMY. The cone of influence (COI) in which marginal effects should be considered, is blurred. Note that the wavelet scales in the diagrams are converted to approximate frequencies.
